# Function of *Akkermansia muciniphila* in Obesity: Interactions With Lipid Metabolism, Immune Response and Gut Systems

**DOI:** 10.3389/fmicb.2020.00219

**Published:** 2020-02-21

**Authors:** Yu Xu, Ning Wang, Hor-Yue Tan, Sha Li, Cheng Zhang, Yibin Feng

**Affiliations:** School of Chinese Medicine, Li Ka Shing Faculty of Medicine, The University of Hong Kong, Pokfulam, Hong Kong

**Keywords:** *Akkermansia muciniphila*, obesity, lipid modulation, nutrition therapy, immunity

## Abstract

Obesity and its metabolic syndrome, including liver disorders and type 2 diabetes, are a worldwide epidemic and are intimately linked to diet. The gut microbiota interaction has been pointed to as a hot topic of research in the treatment of obesity and related metabolic diseases by influencing energy metabolism and the immune system. In terms of the novel beneficial microbes identified, *Akkermansia muciniphila* (*A. muciniphila*) colonizes the mucosa layer of the gut and modulates basal metabolism. *A. muciniphila* is consistently correlated with obesity. The causal beneficial impact of *A. muciniphila* treatment on obesity is coming to light, having been proved by a variety of animal models and human studies. *A. muciniphila* has been characterized as a beneficial player in body metabolism and has great prospects for treatments of the metabolic disorders associated with obesity, as well as being considered for next-generation therapeutic agents. This paper aimed to investigate the basic mechanism underlying the relation of *A. muciniphila* to obesity and its host interactions, as identified in recent discoveries, facilitating the establishment of the causal relationship in *A. muciniphila*-associated therapeutic supplement in humans.

## Introduction

Gut microbes have been reported to play a vital role in the regulation of host metabolism in both human and animal studies (Sonnenburg and Backhed, 2016). Host intestine microbiota comprising tens of trillions of microorganisms and more than 1000 different bacteria species have been identified, with at least 3 million genes. The species classification of gut community members is enormous because the distribution and composition of gut microbiota are varied at different intestine anatomical sites and are prone to be influenced by external and internal factors, including lifestyle, diet, and body condition (Clemente et al., 2012). The layout and dysbiosis of gut microbiota have been described to have close pathological and physiological interactions with obesity and metabolic syndrome (Carding et al., 2015). For example, Leptin^ob/ob^ obese mice exhibited a significant decrease in the level of *Bacteroidetes* and a proportional increase in *Firmicutes* abundance in intestine bacteria, which spurred further investigation of the link between the gut microbiota and obesity (Ley et al., 2005). In subsequent studies, WT C57BL/6J mice were fed either a chow diet or a western diet. It was demonstrated that diet-induced obesity could lead to a marked decrease in some *Bacteroidetes* spp. and increased numbers of *Firmicutes*, which was consistent with the observation in the genetic obese animal model (Turnbaugh et al., 2008). A high-fat diet (HFD) could cause shifts in the abundance of dominant gut microbiota, including the phylum *Proteobacteria* as well as *Firmicutes*, followed by *Lactobacillus gasseri* and *Bifidobacterium* spp., both of which were less abundant (Wang and Jia, 2016). In human studies, a lower population of *Bacteroidetes* in combination with a higher proportion of *Firmicutes* than in lean control has been detected in obese patients (Nehra et al., 2016). Interestingly, these changes could be reversed by bariatric surgery or dietary intervention. More specifically, the gut microbiota has been proved to have a necessary capacity to harvest energy from the diet and take action on the pathophysiology of obesity via regulating glucose or lipid metabolism (Sonnenburg and Backhed, 2016). Particularly, *Akkermansia muciniphila* (*A. muciniphila*) found in the healthy human fecal specimen, has been shown to be one of the few dominant bacteria that have been intensively implicated in the development of obesity (Ottman et al., 2017b).

*A. muciniphila* has been classified as the sole gram-negative emblematic *Verrucomicrobia* (Belzer and De Vos, 2012), which is extensively present in the human intestinal mucosa (Huang et al., 2015). Since Derrien et al. (2004) identified the type strain of *A. muciniphila* as MucT (ATCC BAA-835 1/4 CIP107961T) (Zhai et al., 2018), gene sequencing analysis has shown that there are a lot of mucinase-encoding gene candidates and that its single chromosome contains 2176 genes with a 55.8 percentage of GC content. This oval-shaped and non-motile microorganism is a strict anaerobe and chemo-organotroph that can tolerate low oxygen concentrations (Brodmann et al., 2017). *A. muciniphila* was capable of producing mucin-degrading enzymes and utilizing mucins as a nitrogen and carbon source in the mucus layer of epithelium. *A. muciniphila* decomposed these substances into acetic and propionic acid (Huang et al., 2015; Ottman et al., 2016, 2017a) and released sulfate via mucin fermentation. Based on the analysis of its characteristic 16SrRNA signature, *A. muciniphila* constitutes 3 to 5 percent of the gut microbial community in healthy adults, but this level varies according to many factors. *A. muciniphila* has shown a close relationship with age in healthy humans. Its colonization starts from early childhood and reaches a similar level to adults, ranging from 5.0 to 8.8 log cells/g, in a year, but it is reduced in the elderly (Collado et al., 2007, 2008; Wang, 2011). Moreover, The presence of *A. muciniphila* and the mucosal pathology differed in patients with metabolic diseases, and they have been inversely associated with the severity of appendicitis and inflammatory bowel disease (Swidsinski et al., 2011). Moreover, the level of gut *A. muciniphila* showed a negative correlation with diabetes, obesity, and other metabolic syndromes (Dingemanse et al., 2015; Remely et al., 2016; Sergey et al., 2017). The dual regulation between *A. muciniphila* and metabolic diseases has shown that not only can the abundance of *A. muciniphila* be influenced by the presence of pathology but also supplementation of *A. muciniphila* can have effects on the host body. The propagation of *A. muciniphila* in the early gut microbiota colonization by early life treatment with vancomycin could have beneficial effects on controlling the development of autoimmune diabetes (Hansen et al., 2012). Oral administration of *A. muciniphila* at the dose of 2 × 10^8^ bacterial cells per day could reverse HFD-induced obesity in mice by mediating adipocyte metabolism and gut barrier function without influencing food intake (Cani and de Vos, 2017). This preliminary research provided essential evidence that *A. muciniphila* may be considered a promising new therapeutic agent for obesity. At present, the majority of research has demonstrated the beneficial impact of *A. muciniphila* in the prevention and amelioration of metabolic disorders and obesity. And type 2 diabetes mellitus (T2DM) has been characterized as featuring a lower level of *A. muciniphila*, low-grade inflammation, and gut permeability disruption (Pascale et al., 2019). The enrichment degree of *A. muciniphila* can be used as an indicator to evaluate body metabolic status, including glucose homeostasis, serum lipids, and the adipocyte distribution in human (Dao et al., 2016). However, the detailed mechanism of the association between *A. muciniphila* and obesity development has not yet been completely elucidated. Hence, we have reviewed the latest research concerning the role of *A. muciniphila* in obesity and gained an insight into its action on distinctive expression changes of pathways involved in metabolic homeostasis.

## Methods

We made a comprehensive search of research and reviews in journals about gut microbiota and obesity, but not conference papers or reports. When we entered our school library website, the library provided the most relevant databases containing a wide range of key terms including gut microbiota, obesity, human, and *A. muciniphila*. Both the United States National Library of Medicine National Institutes of Health (PubMed) and the Web of Science databases include publications from major academic publishers like Elsevier, Springer, Taylor and Francis, etc. We searched both on PubMed and the Web of Science, retrieved manuscripts and removed duplicate manuscripts. Based on the title and abstract, we excluded some unrelated citations and retrieved 146 full-text articles for further evaluation.

## Results

### Influence of *Akkermansia muciniphila* on Lipid Desregulation in Obesity

Energy consumption promotion has been considered an effective method for body weight loss. Pharmacotherapy, diet, and lifestyle intervention improve energy metabolism, of which the related mechanisms have been proved to involve interaction with gut microbiota (Zhang et al., 2019). As gut microbiota dysbiosis is a contributing factor to energy imbalance, gut microbiota intervention is a potential therapeutic method for treating obesity-related metabolic diseases, including hyperlipidemia and hyperglycemia (Kim et al., 2019). The relationship between *A. muciniphila* and the host is reflected in the energy consumption associated with glucose and lipid metabolism (Zhang et al., 2019) and thereby affecting the development of obesity. Previous studies proved a close negative link between the enrichment of *A. muciniphila* and the development of obesity, in that its abundance is inversely proportional to the bodyweight of animals and humans. Replenishment with *A. muciniphila* in obese mice reversed high fat diet-induced metabolic disorders such as metabolic endotoxemia, fat-mass gain, and insulin resistance (Everard et al., 2013). However, oral administration of *A. muciniphila* did not change plasma lipid in normal chow diet-fed mice (Li et al., 2016). This observation indicated that *A. muciniphila* only showed its modulation effect in the context of lipid metabolic disorder. In cAMP-responsive binding protein H (CREBH) deficiency-induced hyperlipidemia mice, *A. muciniphila* inoculation could cause clearance of triglyceride and postprandial chylomicrons to avoid acute lipid overload in the circulation. During the clearance progress, the expression of low-density lipoprotein (LDL) receptors was increased, which facilitated the upregulation of intermediate-density lipoprotein (IDL) via the induction of apolipoprotein B 100 and apolipoprotein E (Shen et al., 2016; Bannerman et al., 2018). Thus, replenishment with *A. muciniphila* can ameliorate western diet-induced atherosclerotic lesion aggravation in Apolipoprotein E deficient (Apoe^–/–^) mice. Although the colonization of live *A. muciniphila* did not result in the alteration of serum total cholesterol (TC), total triglyceride (TG), high density lipoprotein (HLD), and LDL in Apoe^–/–^ mice, the soluble receptor for tumor necrosis factor type II (sTNFRII) was significantly reduced, which led to improvement in atherosclerosis and congestive heart failure (Li et al., 2016). The metabolic activity of *A. muciniphila* on the host metabolic physiology has also been determined to interact directly with several lipid metabolic substances, including changing the endotoxin level (Ozkul et al., 2017) and short-chain fatty acid (SCFA) production (Ottman et al., 2017b), as well as increasing fatty acid oxidation in the intestine and adipose tissue (Lukovac et al., 2014). As a producer of SCFAs, *A. muciniphila* has been reported to convert dietary fiber into acetate, propionate, and butyrate; these metabolites can have effects on glucose and lipid homeostasis (Chambers et al., 2018). The SCFA production in the distal ileum induced the action of the various transcription factors to control lipid metabolism and growth; for example, propionate and butyrate stimulation led to an obvious increase in fasting-induced adipocyte factor (Fiaf) production and decreased Gpr43, histone deacetylase (HDAC), and PPARγ expression. All these three SCFAs (acetate, propionate, and butyrate) significantly increased Hdac3 and Hdac5 production in intestinal epithelial organoids (Lukovac et al., 2014). The recognition of the beneficial impact of *A. muciniphila* on metabolic disorders during obesity provoked the further investigation of internal cytokines or external metabolites to make a clear statement on the metabolic activities of *A. muciniphila*, particularly with regards to some novel bioactive substances (Schneeberger et al., 2015). For example, compared with T2DM patients, an increased level of *A. muciniphila*-derived extracellular vesicles (AmEVs) was found in the fecal samples from healthy human. Meanwhile, AmEVs reduced body weight, improved glucose tolerance, and ameliorated gut permeability in HFD-induced diabetic mice, as well as enhancing tight junctions via upregulating the expression of occludin in lipopolysaccharide (LPS)-induced Caco-2 cells (Chelakkot et al., 2018). Since *A. muciniphila* has been considered to be a promising prebiotic in the improvement of metabolic syndrome, viable *A. muciniphila* have been implemented for metabolic disorder treatment (Ouwerkerk et al., 2017). Pasteurization is an effective way to make *A. muciniphila* safe for use. Researchers have found that pasteurization enhanced the capacity of *A. muciniphila* to reduce fat mass and improve dyslipidemia (Plovier et al., 2017; Anhe et al., 2019). This enhancement had a close connection with the host intestinal energy metabolism and promoted its capacity to reverse the high expression of Fmo3, which modulated TMA conversion into TMAO. In more detail, this action was associated with the interaction of Toll-Like Receptor 2 signaling via Amuc_1100, a specific protein located at the outer membrane of *A. muciniphila* (Plovier et al., 2017). Amuc_1100 has been proved to modulate the gut barrier and intestinal permeability via tight-junction proteins such as Occludin (Ocln), Claudin 3 (Cldn3), and Cannabinoid Receptor 1 (Cnr1) (Li et al., 2016).

### The Causative Role of *Akkermansia muciniphila* in Liver Disorder in Obesity

**FIGURE 1:**
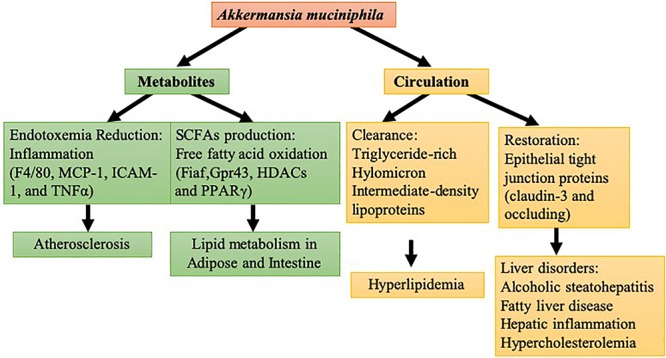
Molecular modulation of *A. muciniphila* in lipid and liver disorder: in yellow are the circulation mechanisms of *A. muciniphila* in hyperlipidemia and liver disorder. In green are the metabolite mechanisms of *A. muciniphila* in lipid metabolism in tissue and atherosclerosis.

Liver disorder disease is a complicated metabolic disease and commonly shows a close relationship with obesity as well as the related gut microbiota changes in obesity (Duranti et al., 2017). In gut microbiota transplantation studies, germ-free C57BL/6J mice receiving intestinal bacteria from HFD-fed mice with high blood glucose were prone to develop liver steatosis and insulin resistance compared with counterparts that were transplanted with bacteria from healthy mice (Aron-Wisnewsky et al., 2013). Different degrees of liver pathology have also been linked with the different varieties of gut microbiota (Meng et al., 2018). Human studies have indicated that the abundance of *Bacteroidetes* in non-alcoholic fatty liver disease patients is much lower than that in simple steatosis and healthy groups (Fukui, 2015). Compared with healthy adults, an apparent decline has been shown in the abundance of *A. muciniphila* in patients with alcoholic steatohepatitis (Van Best et al., 2015). Collective findings have confirmed that the action of *A. muciniphila* could extend to various hepatic disorders such as fatty liver disease, hepatic inflammation, and hypercholesterolemia. Oral supplementation of *A. muciniphila* can restore *A. muciniphila* depletion caused by ethanol exposure in mice, preventing the liver from alcoholic injury, neutrophil infiltration, and steatosis (Grander et al., 2018). In a concanavalin A-induced liver injury mouse model, oral administration of *A. muciniphila* reduced serum Alanine Aminotransferase (ALT) and Aspartate Aminotransferase (AST) and alleviated liver damage (Wu et al., 2017). Mechanistic study revealed that *A. muciniphila* improved epithelial barrier function via fluorescein isothiocyanate-dextran translocation (FD4) in mucosa to elevate gut status. *A. muciniphila* treatment restored epithelial tight junction proteins (claudin-3 and occludin) in colonic epithelial cells to defend against the invasion of ethanol. The evidence suggests that *A. muciniphila* has protective actions against alcoholic liver disease focusing on the intestine (Grander et al., 2018). Moreover, the interference of *A. muciniphila* in improving intestinal barrier function is evoked by improving occludin and Tjp-1 expressions and suppressing LPS production followed by increasing the variety and volume of gut microbes (Wu et al., 2017). Above all, as shown in Figure 1, during the development of lipid and liver disorders, *A. muciniphila* modulated the lipid metabolism in circulation, including adipose, liver, and intestine, and the internal metabolite changes caused by *A. muciniphila* were also involved in these actions.

### Regulation of the Inflammatory Response in Obesity by *Akkermansia muciniphila*

Obesity and associated metabolic disorders are characterized by a low-grade inflammatory response. Previous reports have documented that antibiotic or probiotic carbohydrate treatment modulated gut microbiota, leading to a reduction in metabolic endotoxemia that induced low-grade inflammation. The anti-inflammatory process is associated with lower intestinal permeability in diet-induced obesity mice via improving the tight-junction integrity (Cani et al., 2008), and it has been confirmed that this process might control and increase the production of endogenous GLP-2 in Leptin^ob/ob^ mice (Cani et al., 2009). The intervention of *A. muciniphila* in germ-free mice suggested the existence of host-*A. muciniphila* interaction in obesity involving the activation of immunological signaling (De Vos, 2017). This interaction is supported by evidence indicating that *A. muciniphila* was highly enriched in Rag1^tm1Mom^ mice deficient in B and T lymphocytes compared with wild-type mice (Zhang et al., 2015). *A. muciniphila* has been proved to have a robust correlation with inflammatory markers and adipose tissue homeostasis, and also with insulin and glycemia at the onset of obesity (Schneeberger et al., 2015). The indirect alteration evoked by *A. muciniphila* in obesity also involved the stimulation of Treg cell proliferation (Shin et al., 2014). The triglyceride clearance action of *A. muciniphila* in CREBH-null mice was mediated by LDL receptor signaling, which further involved the suppression of hepatic ER stress marker-glucose regulated protein 94 and inflammatory transcription factors Jun-amino-terminal kinase 1 (JNK1) and JNK2 (Shen et al., 2016). Accumulating evidence concerning the correlation between gut permeability and LPS absorption has provided reliable support the identified association with metabolic inflammation (Cani et al., 2012). *A. muciniphila* restored the gut barrier function via decreasing LPS evoked by a western diet (Kelly et al., 2015). The amelioration of LPS-induced artic and systemic inflammation suppressed the macrophage transmigration into intima and adhesion onto endothelium, as evidenced by the reduced expressions of ICAM-1, MCP-1, F4/80, TNF-α, and IL-1β (Anonye, 2017; Wang et al., 2017). Colonization of *A. muciniphila* in an immune-mediated liver injury model restrained the increased LPS level and substantially decreased the expression of serum pro-inflammatory markers (interleukin(IL)-2, Interferon-γ (IFN-γ), IL-12p40, MCP-1, MIP-1a, and MIP-b), as well as attenuating the hepatocellular apoptosis by the Toll-like receptor-4 (TLR-4) pathway (Wu et al., 2017). MyD88/TLR2 innate immune signaling also appeared to be mediated by *A. muciniphila*, and *A. muciniphila* consistently evoked a lower level of serum endotoxin and islet toll-like receptor, as well as promoting the secretion of antimicrobial peptide Reg3γ in colon, which caused bacterial remodeling in non-obese diabetic mice (Hanninen et al., 2018). Interferon-γ (IFNγ), as a key cytokine of the immune system in glucose metabolism, could be negatively modulated by *A. muciniphila*, which led to the improvement of glucose tolerance. The IFNγ-modulated gene Irgm1 has been identified in the mouse ileum and can regulate the abundance of *A. muciniphila* (Greer et al., 2016). However, *S.* Typhimurium-induced inflammation could turn the commensal bacterium into a pathobiont. *A. muciniphila* can exacerbate *S.* Typhimurium-induced inflammation, coinciding with higher mRNA levels of IFNγ and IFNγ-induced protein 10 (IP-10), tumor necrosis factor-α (TNF-α), and interleukin (IL)-6 and IL-17, but not IL-18. This inflammatory exacerbation was found in a gnotobiotic mouse model colonized with a defined simplified microbiota of eight bacterial species (Zhai et al., 2015; Pleasure et al., 2018). The presence of *A. muciniphila* enhanced the infection with *S.* Typhimurium and promoted macrophage infiltration into cecal tissue. *A. muciniphila* at extremely high cell concentrations of 10^6^ bacteria/ml can stimulate interleukin 8 (IL-8) secretion on enterocytes. This means that the low level of pro-inflammatory stimulation evoked by *A. muciniphila* seemed unable to insult a powerful inflammatory cascade. Conversely, the low-level stimulation involved LPS production may keep the mucosa-associated immune system alerted at an appropriate level (Reunanen et al., 2015). It is likely that, normally, *A. muciniphila* shows a protective role in intestinal immunity but that the role of *A. muciniphila* in the ecosystem can be changed in response to the presence of inflammation (Ganesh et al., 2013). Despite the fact that there is no clear definition of the appropriate levels that can describe a shift between a pathological and a healthy condition, below a given amount of *A. muciniphila*, human subjects were less likely to have a positive response in terms of improved inflammatory cytokines and insulin resistance (Schneeberger et al., 2015). In contrast to the adverse effect of *A. muciniphila* in the development of DSS-induced colitis, *A. muciniphila*-derived extracellular vesicles, as a mediator on intestine immunity, have shown an obvious protective function against the progression of DSS-induced colitis (Kang et al., 2013). To some degree, the host regulation of *A. muciniphila* contributed to its derived materials, including pili-like proteins (Ottman et al., 2017c). The outer membrane pili-like protein of *A. muciniphila* (Amuc_1100) directly modulated the gut immunity and increased trans-epithelial resistance, which enhanced the capacity of *A. muciniphila* to improve host metabolic homeostasis (Ottman et al., 2017c).

### Interaction of *Akkermansia muciniphila* With the Gut Ecosystem and Homeostasis in Obesity

**FIGURE 2:**
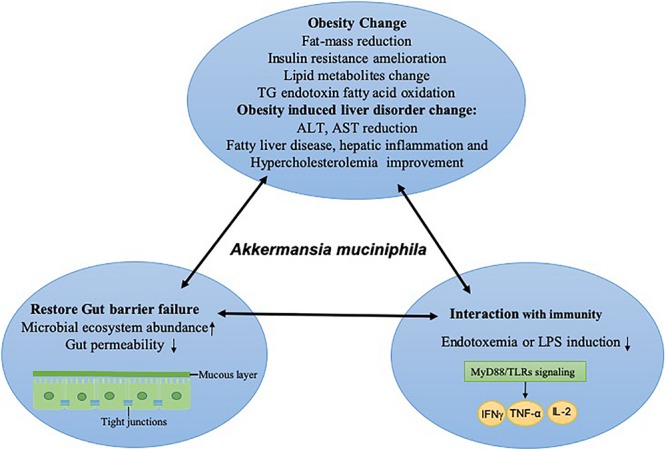
Cross talk in host-*A. muciniphila* interactions and the associated improvement in obesity disease. After the colonization of *A. muciniphila*, the state of homeostasis and symbiotic relationships is improved by immunity activity. This state includes obesity and its associated liver disorder changes in the host; the gut barrier is restored either physically (at the mucous layer or the level of tight junctions) or at the level of the gut ecosystem, and reduced inflammatory responses are often induced by endotoxemia or LPS via TLRs and downstream cytokines, which improve intestinal permeability. TG, total triglyceride; ALT, Alanine Aminotransferase; AST, Aspartate Aminotransferase; LPS, lipopolysaccharide; TLR, Toll-like receptors; IFNγ, Interferon-γ; TNF, tumor necrosis factor, IL-2, interleukin-2.

Certainly, to determine whether the gut microbiota contributes to maintaining a healthy status, the variation in the host micro-environment ecosystem should not be overlooked (Lin et al., 2014). Poor diversity of the gut ecosystem can result in the alteration of intestinal permeability and the breakdown of the intercellular junctions between mucosal cells, both of which permit the diffusion and activation of potentially immune substances from intestinal content into an inflammatory state, causing a disorder of the gut tract. It has been reported that mucins constitute a key ecological niche that shapes the microbiota composition. Supplementation of mucins had a positive impact on the abundance of *Akkermansia, Bacteroides*, and *Ruminococcus* (Van Herreweghen et al., 2018), while higher simulated colonic pH showed less positive effects on the level of *Akkermansia*. In addition to the nutritional resources and environmental factor modulation on the gut microbiome, the extracellular mucin degradation by *Akkermansia* could provide growth benefits to gut community species via trophic interactions (Belzer et al., 2017; Zhang S. et al., 2018). The mucolytic activity of *A. muciniphila* could produce mucin-degrading enzymes (proteases, glycosyl hydrolases, and sulfatases), oligosaccharides and short-chain fatty acids (SCFAs, including lactate and acetate), all of which juxtaposed community members could utilize for their own proliferation. For example, the mucolytic enzymes induced by *A. muciniphila* increased upon the presence of a butyrogenic gut commensal, *Anaerostipes caccae* (Chia et al., 2018). Furthermore, the monosaccharides supported by *A. muciniphila* promoted the proliferation of *Faecalibacterium prausnitzii*, which converted acetate and lactate into butyrate (Lopez-Siles et al., 2018). It has been shown that *A. muciniphila* and *F. prausnitzii*, co-occurring in the mucosa, had a syntrophic relationship with each other and that both species were reduced in inflammatory bowel disease (Lopez-Siles et al., 2018). The metabolites were induced by *A. muciniphila*, even though not completely, and thus created metabolic cross-feeding interactions in the gut ecosystem. Moreover, the enrichment of *A. muciniphila* promotes microbial gene richness and microbial ecosystem abundance, including *Firmicutes, Bacteroidetes, Actinobacteria*, and *Euryarchaeota* (Dao et al., 2016). *A. muciniphila* can form an alliance with *Bacteroides-Prevotella*, *Bacteroidetes, Firmicutes*, and *Lactobacillus*, so after the colonization of *A. muciniphila*, the elevated abundance of *A. muciniphila* and these allies indicates that the protective effects have also altered gut microbe community composition. The colonization of *A. muciniphila* in mice also evoked a global transcriptional host response, which revealed a unique, consistent, and site-specific regulation of 750 genes (Derrien et al., 2011). The intestinal epithelium is the mediator of gut microbe and gut barrier function, and the gut barrier status is directly reflected by mucus layer alteration and the changes in immune mediators secreted by host tissue. The interaction with colonic mucin caused by *A. muciniphila* has been elevated at the mucin microarray platform, and it had shown strong mucin binding capacity compared with *Desulfovibrio* spp. (Earley et al., 2015). Specifically, oral *A. muciniphila* administration as a prebiotic treatment for diet-induced obesity mice not only increased Muc2 protein production to restore mucus layer thickness but also prevented the intestinal barrier from injury via decreasing high fat-induced endotoxemia (Everard et al., 2013). *A. muciniphila* is located in close relation to the intestinal cells. Its adherence to the enterocyte cells in the intestinal tract strengthens the integrity of this epithelial cell layer, resulting in the activation of the intestinal immune system and resistance to leaky gut (Derrien et al., 2011; Reunanen et al., 2015). Moreover, *A. muciniphila* reduced gut permeability by improving the inner mucin layer thickness of ileum in Apoe^–/–^ mice, which involved increased expressions of Occludin and ZO-1 (Li et al., 2016). In addition, the strong adhesion of *A. muciniphila* to Caco-2 and HT-29 human colonic cell lines helped to strengthen the integrity of enterocyte monolayer via increasing the transepithelial electrical resistance (Reunanen et al., 2015). *A. muciniphila* can secrete some enzymes into the gut tract to regulate the mucin protein on the mucosa layer (Geerlings et al., 2018). The related microbiota on the mucosa layer and its associated composition have beneficial effects on protecting the intestine epithelial cells from injury. Cross-talk in gut-*A. muciniphila* interactions and the associated improvement in obesity disease are shown in Figure 2. It has been shown through pathway reconstructions that alteration of mucosal gene profiles by colonization by *A. muciniphila* is involved in an increase in expression immune response- and cell fate determination-related genes (Derrien et al., 2011). The colonization of *A. muciniphila* in germ-free mice altered the mucosal gene expression, according to the immune pathway reconstruction, which indicated that the pathways modulated by *A. muciniphila* involved mucus homeostasis for immune tolerance toward commensal microbiota and basal metabolism (Derrien et al., 2011).

## Nutritional and Medicinal Approches for Modulation of *Akkermansia muciniphila*

As the major environmental factor for intestinal microbiota, dietary intervention, as a healthy lifestyle approach to treating obesity, has been proved to be beneficial for intestine homeostasis and to be strongly associated with a remarkable change in *A. muciniphila* abundance in the gut (Anhe et al., 2016). Dietary interventions for reducing obesity have been suggested that focus on calorie restriction, energy reduction, or a diet rich in prebiotic fibers, all of which are associated with an increased level of *A. muciniphila* in the host (Verhoog et al., 2019). For example, clinical trials have shown a significant increase in *A. muciniphila* in a calorie restriction study of 49 overweight and obese adults (Stenman et al., 2016). Diets rich in prebiotic fiber increased the number of *A. muciniphila* in the microbiome (Gomez-Gallego et al., 2016). Meanwhile, direct probiotic supplements can cause a >100-fold enrichment in the level of *A. muciniphila* (Everard et al., 2013; Zhao et al., 2017). Pomegranate extract promoted the growth of *A. muciniphila in vivo* and led to the formation of ellagic acid (Wang et al., 2016; Henning et al., 2017). Furthermore, a review indicated that the metabolic benefits of polyphenol-rich extracts, such as cranberry extract, curcumin, and epigallocatechin gallate, contribute to the prevention of weight gain and obesity-related metabolic disturbances through increasing the abundance of this bacterial species in the gut microbiota. In line with these findings, the molecular mechanism by which cranberry extract increased mucin 2 secretion has been explored; it might supply ample trophic resources for *A. muciniphila* to thrive and might lead to improved gut mucous layer in mice (Pierre et al., 2013). Moreover, a butter fat-based diet supplemented with California table grape powder can reduce obesity and hepatic lipogenesis in mice, associated with an increase in *A. muciniphila* in the gut (Baldwin et al., 2016). A higher abundance of *A. muciniphila* caused by the anti-obesity effects of Capsaicin also induces the increased expression of anti-microbial protein Reg3g and Mucin 2 protein in the gut (Shen et al., 2017). Compared with healthy patients, metformin promoted the abundance of *A. muciniphila* in diabetic patients. *A. muciniphila* further contributed to the anti-diabetic effects of metformin via enrichment of mucin degradation and SCFA secretion in type 2 diabetes patients (De La Cuesta-Zuluaga et al., 2017). Dietary polyphenols have shown protection against HFD-induced obesity through modification of the gut microbiota community. Oral administration of a steroidal saponin extract from *Agave salmiana* in HFD mice showed anti-obesity effects, attenuating hepatic steatosis, and increased the population of *A. muciniphila* in a dose-dependent manner (Leal-Diaz et al., 2016). Dietary polyphenols resulted in a reduced inflammatory response in the intestine and led to an improvement of metabolic syndrome. Other dietary polyphenols such as cranberry polyphenol extract have shown anti-obesity effects associated with promoting the growth of *A. muciniphila* via downregulation of inflammatory cytokines such as TNF-α and IL-6 (Roopchand et al., 2015). However, the mechanism underlying the effect of these polyphenols in promoting the abundance of *A. muciniphila* is still unclear, and further studies are needed to confirm whether these effects on gut microbiota are mediated or direct (Spanogiannopoulos et al., 2016). However, it has recently been proved that black tea- or grape-derived polyphenols can directly upregulate the expansion of *A. muciniphila* (Etxeberria et al., 2015), further work is needed to find the underlying mechanism involved (Ozdal et al., 2016). In conclusion, the content of *A. muciniphila* in the microbiome can be enhanced by medical and nutritional therapy, including diet, medicine, prebiotics, and probiotics (Duparc et al., 2017), The ease of *A. muciniphila* supplementation through nutritional therapy indicates that these actions might be an effective method for the treatment of metabolic diseases. Still, not all medicinal nutrition can cause increased *A. muciniphila*, for example, grape proanthocyanidins do not (Zhang L. et al., 2018).

## Discussion and Conclusion

**FIGURE 3:**
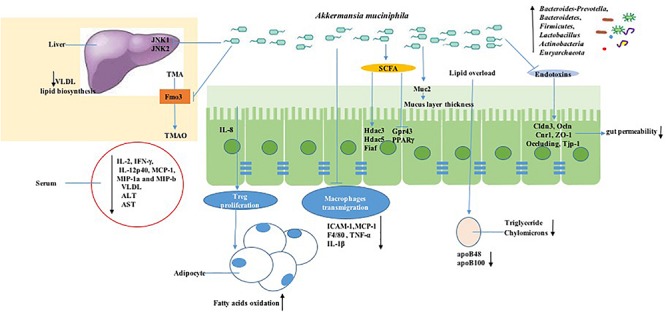
Mechanisms of signaling due to *A. muciniphila* action in obesity. *A. muciniphila* can decrease the serum level of inflammatory cytokines such as IL-2, IFN-γ, IL-12p40, and MCP-1. Meanwhile, *A. muciniphila* decreases the lipid overload process associated with the LDL receptor pathway by decreasing apoB48 and apoB100 on LDLs. Moreover, short-chain fatty acid production stimulated by *A. muciniphila* is involved in signaling to the host by inhibiting histone deacetylase (HDAC) or by activating G-protein-coupled receptors, which triggers other metabolic pathways, together resulting in immune stimulation including macrophage transmigration and Treg proliferation changes. *A. muciniphila* regulates the intestinal permeability and gut barrier by tight-junction proteins such as Claudin 3 (Cldn3), Occludi(Ocln), and Cannabinoid Receptor 1 (Cnr1) and reverses the high enzyme expression of flavin-containing monooxygenase 3 (FMO3), which modulates TMA conversion into TMAO in the liver.

Consistent findings from both preclinical and clinical research have revealed declined abundance of *A. muciniphila* in obesity and metabolic syndromes. With more preclinical and clinical research focused on *A. muciniphila*, the molecular mechanism of *A. muciniphila* for treating metabolism is becoming more apparent. It is worth looking forward to finding the systematic regulatory mechanism of *A. muciniphila* in rodents to provide essential evidence for human research; hopefully, these achievements can be translated to human intervention research, though human studies still hold uncertain ethical and inevitable practical limitations. From the schematic overview of the metabolic activities of *A. muciniphila* in intestine and interaction with the host as a result of *A. muciniphila* colonization (Figure 3), conclusions can be drawn. Oral gavage with *A. muciniphila* or the beneficial dietary polyphenol increased the abundance of intestinal *A. muciniphila* to a level sufficient to reduce systemic inflammation, improve gut disruption, and contribute to the steady state of the host basal metabolism and the promotion of other beneficial microbes via various signaling pathways. This suggests that therapeutic intervention focusing on *A. muciniphila* in intestinal microbiota could be considered as a promising strategy for the prevention and treatment of obesity and metabolic disorder diseases, including cardiovascular disease, liver injury, and type II diabetes mellitus (Zhang L. et al., 2018). These statements can be supported by the evidence, as shown in Table 1, which shows an overview of *Akkermansia* and its associated biomarkers in clinical studies of obesity. A recent clinical trial (NCT02637115) indicated that 3-month oral administration of *A. muciniphila* in obese patients is safe and well-tolerated. Meanwhile, supplementation of *A. muciniphila* decreased the body weight and improved liver dysfunction as well as inflammation in patients, as shown in a double-blind, randomized human study (Depommier et al., 2019). It has been demonstrated that *A. muciniphila* could be isolated from purified hog gastric mucus (Van Der Ark et al., 2018) and cultured *in vivo* for medicinal application. However, the development of translational approaches is limited by a lack of specific growth-dependent compounds and suitable culture conditions without oxygen influence (Ouwerkerk et al., 2016). Another reason that the products, including *A. muciniphila*, are unable to be commercially viable is that their safety assessment cannot reach the requirements of Novel Foods Regulation (Gomez-Gallego et al., 2016). Moreover, though the correlation of gut microbiota alterations and chronic metabolic diseases has been discussed, the consequences or causes of these bacterial changes are still unclear. It is inadvisable to make an absolute statement on the positive effects of *A. muciniphila* on metabolic diseases. For example, an excessive level of *A. muciniphila* has been found to exist in Parkinson’s disease and eczematous infants, and the adverse action of *A. muciniphila* in eczematous infants may cause reduced integrity of the intestinal barrier function, therefore leading to increased risk of eczema development (Zheng et al., 2016). The adverse effect of excessive *A. muciniphila* in Parkinson’s disease is still unclear, but it has been reported that the reduction of SCFAs and the intestinal amyloid deposition evoked by the alteration of gut microbiota are associated with exacerbating cognitive deficits in Parkinson’s disease (Zhang et al., 2017). The gut microbiota has also been shown to have effects on Abeta amyloidosis through the modulation of host innate immunity in Parkinson’s disease (Minter et al., 2017). It is admitted that a shift in microbiota can be influenced by various factors, including environment, species, and age. Therefore, we should conduct a double-blind, randomized, and placebo-controlled pilot study to determine the effects of *A. muciniphila* on rodents or humans and also to ensure experimental reproducibility. Moreover, although we can form a conclusion about the extraordinary achievement made by *A. muciniphila* and understand its molecular mechanism in treating obesity, we are unable to quantify the content of *A. muciniphila* that constitutes an effective dosage (Verhoog et al., 2019). Furthermore, *A. muciniphila* colonization is extensively different in different human groups; for instance, residents of Southern China had a high rate of *A. muciniphila*, and over 12 different subtype strains resided in their guts (Guo et al., 2016).

**TABLE 1:** Overview of *A. muciniphila* and its associated biomarkers in clinical studies of obesity.

**Ref.**	**Participation**	**Intervention or Observation**	**Study Period**	**Results**
(Medina-Vera et al., 2019)	81 Patients with T2DM	A reduced-energy diet	3 months	Dietary portfolio consumption increased levels of *A. muciniphila* and improved glycaemic control, dyslipidaemia, and inflammation.
(Roshanravan et al., 2017)	60 patients, overweight and with obese diabetes	600mg/d butyrate, 10 g/d inulin powder, both inulin and butyrate or placebo	45 days	Supplementation of insulin and butyrate can increase *A. muciniphila*, and butyrate decreases TNF-α mRNA expression, hs-CRP, MDA, and diastolic blood pressure levels.
(Walker et al., 2019)	28 obese men with metabolic syndrome	1 g polyphenol resveratrol orally twice daily or placebo	35 days	Polyphenol resveratrol improves glucose homeostasis and increases the abundance of *A. muciniphila.*
(Allin et al., 2018)	134 Danish adults with prediabetes and 134 healthy controls	Observation		The abundance of the mucin-degrading bacterium *A. muciniphila* obviously decreased in prediabetes.
(Dao et al., 2016)	49 adults, overweight and obese	6-week calorie restriction	12 weeks	*A. muciniphila* abundance improved fasting plasma glucose, plasma triglycerides, and body fat distribution.
(Khan et al., 2018)	43 hypercholesterolemic patients and 19 healthy controls	27 patients with Atorvastatin treatment	2 years	Atorvastatin treatment increased the abundance of *A. muciniphila.*
(Liu et al., 2017)	70 female patients with T2DM and 70 healthy females.	Observation		Decreased *A. muciniphila* was associated with fasting blood glucose and urine glucose.
(Collado et al., 2010, 2012; Santacruz et al., 2010)	16 infants with obese mother and 256 infants of normal mothers as control	Observation		Prevalence of *A. muciniphila* was lower in control infants with normal mothers.
(De La Cuesta-Zuluaga et al., 2017)	28 participants with diabetes and 84 healthy controls	Metformin		Diabetes patients taking metformin had a higher relative abundance of *A. muciniphila* compared with healthy control.
(Palleja et al., 2016)	13 morbidly obese patients	Roux-en-Y gastric bypass (RYGB)	12 months	RYGB changed the relative abundances of 31 species, including *A. muciniphila*, within the first 3 months. These abundance changes can be maintained for 9 months.
(Brahe et al., 2015)	53 women with obesity	Observation		140 metagenomic species, including *A. muciniphila*, were correlated with metabolic markers.
(Yassour et al., 2016; Cortez et al., 2018)	21 patients with T2DM	Duodenal-jejunal bypass surgery medical care	12 months	The level of gut *A. muciniphila* in the surgery group increased.
(Depommier et al., 2019)	32 participants, overweight/obese insulin-resistant	Oral supplementation of 10^10^ A. muciniphila bacteria, either live or pasteurized	3 months	*A. muciniphila* decreased body weight and reduced the levels of the relevant blood markers for liver dysfunction and inflammation, while the overall gut microbiome structure was unaffected.

## Author Contributions

YF conceived and designed the review. YX retrieved the literature and drafted the manuscript. NW, H-YT, SL, and CZ participated in the design of study and modification of English grammar. All authors read and approved the final manuscript.

## Conflict of Interest

The authors declare that the research was conducted in the absence of any commercial or financial relationships that could be construed as a potential conflict of interest.
